# Frontal-posterior functional imbalance and aberrant function developmental patterns in schizophrenia

**DOI:** 10.1038/s41398-021-01617-y

**Published:** 2021-09-27

**Authors:** Dandan Sun, Huiling Guo, Fay Y. Womer, Jingyu Yang, Jingwei Tang, Juan Liu, Yue Zhu, Jia Duan, Zhengwu Peng, Huaning Wang, Qingrong Tan, Qiwen Zhu, Yange Wei, Ke Xu, Yanbo Zhang, Yanqing Tang, Xizhe Zhang, Fuqiang Xu, Jie Wang, Fei Wang

**Affiliations:** 1 Department of Cardiovascular Ultrasound, The People’s Hospital of China Medical University & The People’s Hospital of Liaoning Province, Shenyang, China; 2 Department of Psychiatry, The First Hospital of China Medical University, Shenyang, China; 3 Early Intervention Unit, Department of Psychiatry, Affiliated Nanjing Brain Hospital, Nanjing Medical University, Nanjing, China; 4 Department of Psychiatry, Washington University School of Medicine, St Louis, MO USA; 5 Department of Psychiatry, Xijing Hospital, Fourth Military Medical University, Xi’an, China; 6 Liaoning Key Laboratory of Cognitive Neuroscience, Shenyang Medical College, Shenyang, China; 7 Department of Radiology, The First Hospital of China Medical University, Shenyang, China; 8 Department of Psychiatry, Faculty of Medicine & Dentistry, University of Alberta, Edmonton, Canada; 9 School of Biomedical Engineering and Informatics, Nanjing Medical University, Nanjing, China; 10 Key Laboratory of Magnetic Resonance in Biological Systems, State Key Laboratory of Magnetic Resonance and Atomic and Molecular Physics, National Center for Magnetic Resonance in Wuhan, Wuhan Institute of Physics and Mathematics, Chinese Academy of Sciences, Wuhan, China; 11 Shenzhen Key Lab of Neuropsychiatric Modulation, Guangdong Provincial Key Laboratory of Brain Connectome and Behavior, Brain Cognition and Brain Disease Institute, Shenzhen Institutes of Advanced Technology, Chinese Academy of Sciences, Shenzhen, China; 12 Functional Brain Imaging Institute of Nanjing Medical University, Nanjing, China

**Keywords:** Schizophrenia, Diagnostic markers

## Abstract

Schizophrenia (SZ) is a neurodevelopmental disorder. There remain significant gaps in understanding the neural trajectory across development in SZ. A major research focus is to clarify the developmental functional changes of SZ and to identify the specific timing, the specific brain regions, and the underlying mechanisms of brain alterations during SZ development. Regional homogeneity (ReHo) characterizing brain function was collected and analyzed on humans with SZ (hSZ) and healthy controls (HC) cross-sectionally, and methylazoxymethanol acetate (MAM) rats, a neurodevelopmental model of SZ, and vehicle rats longitudinally from adolescence to adulthood. Metabolomic and proteomic profiling in adult MAM rats and vehicle rats was examined and bioanalyzed. Compared to HC or adult vehicle rats, similar ReHo alterations were observed in hSZ and adult MAM rats, characterized by increased frontal (medial prefrontal and orbitofrontal cortices) and decreased posterior (visual and associated cortices) ReHo. Longitudinal analysis of MAM rats showed aberrant ReHo patterns as decreased posterior ReHo in adolescence and increased frontal and decreased posterior ReHo in adulthood. Accordingly, it was suggested that the visual cortex was a critical locus and adolescence was a sensitive window in SZ development. In addition, metabolic and proteomic alterations in adult MAM rats suggested that central carbon metabolism disturbance and mitochondrial dysfunction were the potential mechanisms underlying the ReHo alterations. This study proposed frontal-posterior functional imbalance and aberrant function developmental patterns in SZ, suggesting that the adolescent visual cortex was a critical locus and a sensitive window in SZ development. These findings from linking data between hSZ and MAM rats may have a significant translational contribution to the development of effective therapies in SZ.

## Introduction

Schizophrenia (SZ) is a neurodevelopmental disorder that typically manifests between late adolescence to young adulthood [1]. Brain alterations in SZ likely reflect both pathological and compensatory processes over a protracted developmental period [2, 3], and further underscore the importance of developmental neuroplasticity in SZ from infancy to adulthood [4]. During this critical period, cortical circuits mature via rearrangement processes, including myelination, synaptic pruning, and dendritic plasticity [5], as well as the maturation of neurotransmitter systems, including the glutamatergic, the dopaminergic, and the GABAergic systems [6]. In SZ, as high-risk states convert to psychosis, neural alterations appear to accumulate and progress from early development onwards, and brain disruptions become widespread and global in adulthood [7, 8]. However, there remain significant gaps in understanding the dynamic brain changes across development in SZ, and it is unclear what are the specific timing, the specific brain regions, and the underlying mechanisms of brain alterations during SZ development.

Animal models may offer important insights to address these knowledge gaps. The major animal models of SZ contained neurodevelopmental, pharmacological, lesion, and genetic models [9, 10]. One such model employs the administration of a mitotoxin, methylazoxymethanol acetate (MAM), during gestation to induce a developmental disruption [11]. At gestational day 17 (E17), through selective disturbance of proliferation and migration of neuronal precursor cells undergoing their final mitosis, MAM induces morphological and cytological alterations on developing paralimbic, frontal, and temporal cortices, regions where deficits are typically observed in SZ patients [12, 13]. Moreover, rats exposed to MAM on E17 have neuroanatomical, neuronal processing, and behavioral alterations similar to SZ, and thus may serve as a neurodevelopmental animal model of SZ [10, 14]. MAM rats have shown reduced size, increased neuronal density, and impaired synaptic activity in the prefrontal and anterior cingulate regions, and reduced size, decreased neuropil, and disordered neuronal cytoarchitecture in the occipital regions [12, 15]. Similarly, significantly decreased gray matter volumes in the orbitofrontal, anterior cingulate, and occipital regions have been found in human with SZ (hSZ) [16]. The similarity of abnormal neuronal synchronization in hSZ and MAM rats has also been observed. Deficits in gamma activity during SZ relevant cognitive tasks and increased background gamma activity have been found in MAM rats, similar to gamma activity patterns observed in hSZ [17, 18]. Interestingly, the functional imbalance between frontal and posterior brain regions has also been found in both MAM rats and hSZ by functional magnetic resonance imaging (fMRI). MAM rats have shown lower functional connectivity (FC) in the orbitofrontal cortex (OFC) and higher FC in the visual cortex, as well as a higher number of long-range connections, and a lower number of short-range connections [19]. Our group previously demonstrated hyperactivity indexed by amplitude of low-frequency fluctuations (ALFF) in the prefrontal cortex (PFC) and hypoactivity in the primary sensory cortices [20], as well as increased medium/long-range connectivity in the PFC and decreased short-range connectivity in the primary sensory cortices [21]. In light of their structural and functional similarities, MAM rats could serve as a neurodevelopmental model for brain organization and function in hSZ.

Several neuroimaging measures have been proposed as potential biomarkers of spatial and temporal dynamics in SZ [22]. Regional homogeneity (ReHo) is a commonly used measure that reflects spontaneous local activity within individual regions. It employs Kendall’s coefficient of concordance (KCC) to measure the temporal similarity of local blood–oxygen-level-dependent (BOLD) signals [23]. ReHo appears dependent on a combination of anatomical, developmental, and neurocognitive factors [24] and has been successfully employed to examine changes or modulations under different conditions across the whole brain in a voxel-by-voxel manner and detect disease-associated regional activity changes [25]. It is a highly sensitive, reproducible, and reliable index in SZ [26], which could serve as a marker of changes in brain dynamics and function in SZ.

This study was conducted in three major steps. Firstly, we identified similar ReHo alterations in adult hSZ and MAM rats. Then, we characterized the longitudinal ReHo changes from adolescence to adulthood in MAM rats. Finally, we examined the metabolomic and proteomic profiles in adult MAM rats to explore the potential mechanisms for the ReHo alterations in SZ.

## Materials and methods

### Study design

**Fig. 1:**
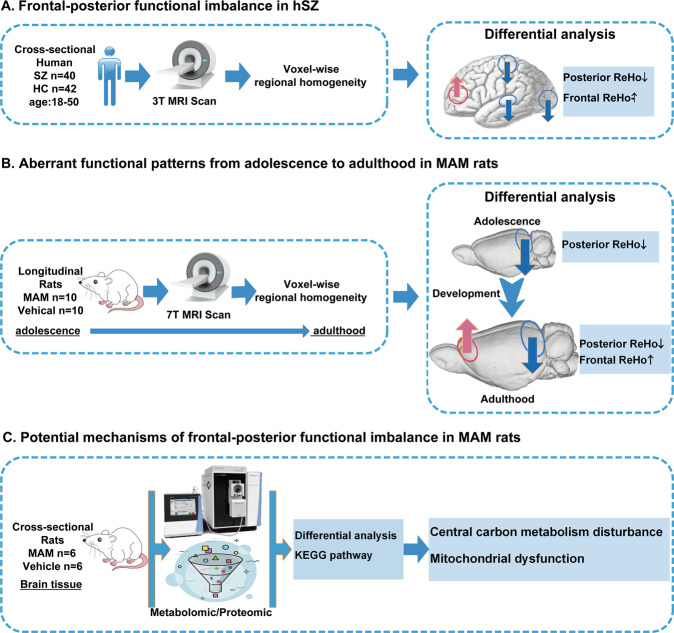
Flow diagram of the study design. **A** We confirmed the frontal-posterior functional imbalance of SZ by comparison of ReHo using cross-sectional fMRI data of hSZ and HC. The frontal-posterior functional imbalance was characterized by increased frontal (frontal cortices) and decreased posterior (visual and associated cortices) ReHo. **B** We determined the aberrant function developmental patterns with respect to the timing and localization of major ReHo changes from adolescence to adulthood using longitudinal design in MAM rats. The aberrant function developmental patterns were characterized by primarily decreased posterior ReHo in adolescence and subsequently decreased posterior and increased frontal ReHo in adulthood. **C** We analyzed brain metabolomic and proteomic profiles from a cross-sectional cohort of MAM rats and vehicle rats. Metabolic and proteomic changes in MAM rats suggested the potential mechanisms underlying the ReHo alterations in adulthood, including central carbon metabolism disturbance and mitochondrial dysfunction. SZ schizophrenia, hSZ human with schizophrenia, HC healthy control, fMRI functional magnetic resonance imaging, ReHo regional homogeneity, MAM methylazoxymethanol acetate, KEGG Kyoto Encyclopedia of Genes and Genomes.

The cohort of human subjects included 40 hSZ with first-episode untreated SZ with illness duration less than two years and 42 healthy controls (HC) between 18 and 50 years old. hSZ were recruited from inpatient and outpatient services in the Department of Psychiatry at the First Hospital, China Medical University and Shenyang Mental Health Center, and HC from the local community of Shenyang. This study was approved by the Institutional Review Board of China Medical University. Written informed consent was obtained from each participant. The absence or presence of Axis I disorder was confirmed using the Structured Clinical Interview for Diagnostic and Statistical Manual of Mental Disorders, Fourth Edition (DSM-IV) via consensus between two experienced psychiatrists. HC did not have current or lifetime Axis I disorders and any first-degree relatives with a history of Axis I disorders. Participants were excluded if had the following conditions: concomitant major medical disorder, neurological disease or head injury with loss of consciousness, and/or substance/alcohol abuse or dependence. Symptoms and cognitive measures were obtained using the Brief Psychiatric Rating Scale (BPRS) and the Wisconsin Card Sorting Test (WCST). fMRI scanning was performed in each participant (Fig. 1A).

The first cohort of animals consisted of 12 MAM rats and 11 vehicle rats. MAM rats or vehicle rats were male offspring of adult pregnant Sprague–Dawley rats (VITAL RIVER Laboratories, Beijing, China) which were randomly administered either 22 mg/kg MAM or 0.9% saline solution intraperitoneally on E17 [27]. Animals were weaned on postnatal day (PD) 21 and rehoused with a density of 2–4/cage. Animals were in 12-h light/dark cycle (lights on 7 am, lights off 19 pm) with the temperature at 21 ± 1 °C and humidity at 30‒70%, and ad libitum access to both food and water. Longitudinal behavioral and fMRI assessments were examined at two developmental points. fMRI was scanned in adolescence (PD21–25) and adulthood (PD63–67). Open field test (OFT) and Morris water maze (MWM) were tested first in adolescence (PD32–39), and then again in adulthood (PD78–85) (Fig. 1B).

The second cohort of animals consisted of six MAM rats and six vehicle rats. Regional brain tissue samples were collected in adulthood (PD73), which were applied for metabolomic and proteomic profiling and analysis (Fig. 1C). All procedures with animals were performed in strict accordance with the guidelines of the Animal Care and Use Committee at Wuhan Institute of Physics and Mathematics, Chinese Academy of Sciences.

### Human MRI data acquisition and processing

All participants underwent fMRI scanning with a GE Signa HD 3.0 T scanner (GE Healthcare, USA) with a standard 8-channel head coil at the First Affiliated Hospital of China Medical University. Functional images were collected using a gradient echo-planar imaging (EPI) sequence with the following parameters: repetition time (TR) = 2000 ms, echo time (TE) = 30 ms, flip angle = 90°; field of view (FOV) = 240 × 240 mm^2^; matrix = 64 × 64; slice = 35; voxel size = 3 mm^3^; slice thickness = 3 mm without a gap. Each participant was instructed to stay awake, close eyes, relax, and minimize thinking during scanning.

All fMRI Images were preprocessed using the Statistical Parametric Mapping 8 (SPM8, http://www.fil.ion.ucl.ac.uk/spm) and Data Processing Assistant for fMRI (DPARSF, http://www.restfmri.net/forum/DPARSF) V2.3 [28]. The first ten time points were discarded due to magnetic saturation effects. The subsequent preprocessing steps included slice time correction and head motion correction. Images from participants with excessive motion (>3 mm of motion and/or 3° rotation) were excluded. Next, the corrected functional images were normalized to Montreal Neurological Institute space using the EPI template in SPM8 and resampled to 3 × 3 × 3 mm^3^. Further, a linear detrend was performed to reduce the influence of increased MRI equipment temperature. Then nuisance regression was carried out with several confounding covariates, including the Friston-24 head motion and the polynomial trend. Finally, temporal band-pass filtering (0.01–0.08 Hz) was applied to the regressed fMRI images.

Individual ReHo maps were generated by calculating the KCC, which was used to measure the similarity of the time series for a given voxel to neighboring 26 voxels in a 3 × 3 × 3 space. KCC values were computed into ReHo *z*-value (zReHo) by subtracting the mean voxel-wise ReHo obtained for the entire brain. All zReHo maps were spatially smoothed with a 6 mm full-width-at-half-maximum (FWHM) Gaussian kernel, generating the final smoothed zReHo (szReHo) maps.

### Animal behavioral assessment

The assessment methods of the OFT and MWM were reported previously [29]. The detailed descriptions were summarized in Supplementary data.

### Animal MRI data acquisition and processing

The rats were anesthetized (2% isoflurane) and secured into a head restrainer with a built-in coil and a body tube. Isoflurane (0.5–1.0%) in a mixture of 30% O_2_/70% N_2_ was delivered through a nose cone and the whole system was placed into the magnet. The respiratory rate and body temperature were monitored using a PC-SAM Small Animal Monitor (SA Instruments, USA). The core temperature of each animal was maintained at 37 ± 0.5 °C with a heating bed. Image acquisition system consisted of a 7.0 T MRI scanner (Bruker Biospin, German) and a rat brain quadrature surface coil (50 mm diameter). fMRI data were acquired using interleaved snapshot EPI, with the following parameter: TR = 1000 ms, TE = 14 ms, matrix size = 64 × 64, FOV = 2.4 × 2.4 cm^2^, slice number = 20, slice thickness = 0.8 mm, and in-plane resolution = 375 × 375 μm. A total of 300 frames were acquired for each experimental run. A Rapid Imaging with Refocused Echoes sequence was used for anatomical scans with TR of 5000 ms, TE of 12 ms, matrix size of 256 × 256, and resolution of 93.75 × 93.75 μm. The other parameters were as that of fMRI.

Preprocessing was performed using Data Processing & Analysis of Brain Imaging (DPABI, rfmri.org/DPABI) V3.0 rat module. A standard anatomical rat template, spatially aligned to a Paxinos–Watson rat brain template, was offered and authorized [30]. First, the voxel dimensions of both anatomical and functional images were scaled by a constant (10) to fit the standard neuroimaging software. Second, skull stripping was operated to the anatomical images of each adolescent or adult rat in order to remove the MRI signal outside the brain. Third, anatomical image of each rat was normalized to the standard anatomical rat template. The anatomical landmarks, including anterior commissure, fornix, posterior commissure, lateral habenular nucleus, aqueduct, paraflocculus, and pyramidal tract, were manually identified in both the template and adolescent or adult rat. If the landmarks did not align between the template and studied rat, the rat was removed. Fourth, functional images were applied with the normalized anatomical image itself. This self-specific normalization is effective in minimizing systematic errors [31]. Then functional images were processed following slice-timing correction, motion correction, spatial normalization, nuisance regression with 24 head motion parameters, and 0.01–0.08 Hz band-pass filter. For each animal and scan, fMRI data without spatial smoothing were used for ReHo calculation. Individual ReHo maps were generated by measuring the KCC. All ReHo maps were computed and standardized into zReHo maps and then dividing the resultant value by the standard deviation. The final szReHo maps were generated by spatially smoothing of zReHo maps with an FWHM of 6 mm.

### Animal metabolomic profiling

On PD73, the brains of rats were obtained and the targeted cortices (prefrontal and occipital cortices) were located and dissected with the guide of brain maps 4.0 [32]. In brief, the metabolites were extracted from the brain samples and injected into an ultra-performance liquid chromatography system (Agilent Technologies, USA) coupled to a quadrupole time-of-flight mass spectrometer (AB Sciex, USA) with electrospray ionization in positive and negative ionization modes. The metabolites were separated using an Acquity BEH C18 column (Waters, Ireland). Total ion current, chromatographic peaks and retention times were obtained. The raw data were converted to MzXML files. Compound identification of the metabolites was performed by precise mass matching (<25 ppm). The secondary spectrum diagram was matched to a self-built database with authentic standards. After filtering and normalizing, the processed data were uploaded into MetaboAnalyst software for further analysis (www.metaboanalyst.ca). The detailed methods of metabolomic profiling were illustrated in Supplementary data.

### Animal proteomic profiling

The methods of sample preparation for proteomic analysis were reported in Supplementary data. The prepared samples were performed on a Q Exactive mass spectrometer that was coupled to Easy nanoLC (Thermo Scientific, USA). Samples were loaded onto a reverse-phase trap column (Thermo Scientific, USA) connected to the C18-reversed-phase analytical column (Thermo Scientific, USA) in buffer A (0.1% formic acid) and separated with a linear gradient of buffer B (84% acetonitrile and 0.1% formic acid). The MS data were analyzed using MaxQuant software version 1.3.0.5 (Max Planck Institute of Biochemistry, Germany). The cutoff of global false discovery rate (FDR) for peptide and protein identification was set to 0.01. Label-free quantification was carried out in MaxQuant. Protein abundance was calculated on the basis of the normalized spectral protein intensity. The mass spectrometry results were forwarded to statistical analysis. The detailed methods of proteomic profiling were illustrated in Supplementary data.

### Statistical analysis

For human fMRI data, SPM8 was used to perform voxel-based two-sample *t* tests to compare ReHo between HC and hSZ, with diagnostic group as an independent factor, and age and sex as covariates. Multiple comparisons were performed using Gaussian random field theory correction (GRF) in DPABI V4.3 (voxel *P* < 0.01, cluster *P* < 0.05). We extracted ReHo value of the clusters that had been shown differences between HC and hSZ. Two-sample *t* tests were performed to determine the differences in ReHo value between HC and hSZ. The significance level was set at *P* < 0.05. To test for inter-group differences in demographic data and clinical characteristics of human participants, two-sample *t* tests or chi-squared tests were performed using SPSS 16.0 statistical software package (SPSS, Chicago, IL). Statistical significance was set at *P* < 0.05.

For animal longitudinal fMRI data, all ReHo maps of vehicle and MAM rats in the two scanned ages (adolescence and adulthood) were then forwarded to two-sample t-tests. Multiple comparisons across voxels were controlled through GRF correction (voxel *P* < 0.05, cluster *P* < 0.05). According to results from ReHo analyses in human and rats, we defined two ROIs. The frontal ROI included three subareas: mPFC, OFC, and ACC, and the posterior ROI included three subareas: visual cortex, auditory cortex, and temporal association cortex. We extracted ReHo value from the overlaps between frontal and posterior ROIs and the clusters identified in voxel-based tests. Post hoc analyses were performed (vehicle in adolescence vs MAM in adolescence, and vehicle in adulthood vs MAM in adulthood) in the two ROIs using two-sample *t* tests. *P* < 0.05 was considered as statistical significance.

For animal behavioral data, the detailed statistical analyses were described in Supplementary data.

For animal metabolomic data, principal component analysis and partial least square discriminant analysis (PLS-DA) were performed for both positive and negative models after log transformation and Pareto-scaling. The variable importance in the projection (VIP) value of each variable in the PLS-DA model was calculated to indicate its contribution to the classification. Metabolites with the VIP value > 1 were further statistical analysis. Two-sample *t* tests were applied to measure the significances of each metabolite between MAM rats and vehicle rats. *P* < 0.05 were considered as statistical significance. KEGG (Kyoto Encyclopedia of Genes and Genomes) pathway analysis based on the differential metabolites was conducted to identify the important disturbed metabolomic pathways between vehicle and MAM rats. Functional pathways with *P* < 0.05 were considered as statistical significance. The FDR was used for *P* values in multiple tests.

For animal proteomic data, two-sample *t* tests were further applied to measure the significance of each protein between vehicle and MAM rats. *P* < 0.05 were considered as statistical significance. Significantly differential proteins were defined as ≥1.5-fold changes. KEGG pathway analysis based on the differential proteins was conducted to identify the important disturbed proteomic pathways between vehicle and MAM rats. Functional categories and pathways with *P* < 0.05 were considered as statistical significance.

## Results

### Frontal-posterior functional imbalance in hSZ

The demographic information and clinical features were presented in Supplementary Table 1. Significant differences were observed in BPRS scores and WCST scores between hSZ and HC (*P* < 0.05).

**Fig. 2:**
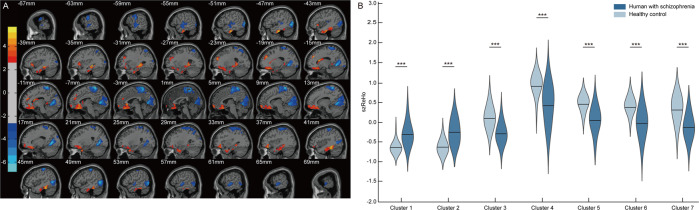
Frontal-posterior functional imbalance in hSZ. **A** Frontal-posterior functional imbalance was found in hSZ compared to HC, expressing as decreased posterior (primary sensory cortices) and increased frontal (higher order frontal cortices) ReHo. **B** ReHo value was extracted from significant clusters identified in the voxel-based test of hSZ vs. HC. Frontal-posterior functional imbalance illustrated as increased frontal ReHo to decreased posterior ReHo from Cluster 1 to Cluster 7. **A** Significant at voxel *P* < 0.01, cluster *P* < 0.05 corrected by GRF correction; **B** significant at *P* < 0.05, *P < 0.05, **P < 0.01, ***P < 0.001. SZ schizophrenia, hSZ human with schizophrenia, HC healthy control, ReHo regional homogeneity.

Frontal-posterior functional imbalance characterized by increased frontal and decreased posterior ReHo were found in hSZ, compared to HC. Significant differences in ReHo were observed in multiple cortical regions (Supplementary Table 2), increased ReHo in heteromodal, limbic, and paralimbic cortical regions, including bilateral dorsal lateral PFC, bilateral OFC, bilateral anterior cingulate cortices (ACC), bilateral paracingulate gyri, bilateral hippocampus, bilateral parahippocampal gyri, bilateral temporal poles, and subcortical regions, including the left caudate nucleus, while decreased ReHo in the primary sensory cortices, including bilateral occipital gyri, bilateral cuneus, bilateral precuneus, bilateral lingual gyri, bilateral calcarine cortices, bilateral temporal gyri, and bilateral postcentral gyrus (*P* < 0.01, Fig. 2A, B).

### Aberrant developmental patterns of brain function from adolescence to adulthood in MAM rats

Generally, compared to vehicle rats, MAM rats exhibited anxiety-like behavior state in adolescence, and impaired spontaneous locomotor activity, anxiety-like behavior state, cognitive deficits in reference memory, and impairment in spatial learning ability in adulthood, which was detailed in Supplementary Figs. 1–3.

**Fig. 3:**
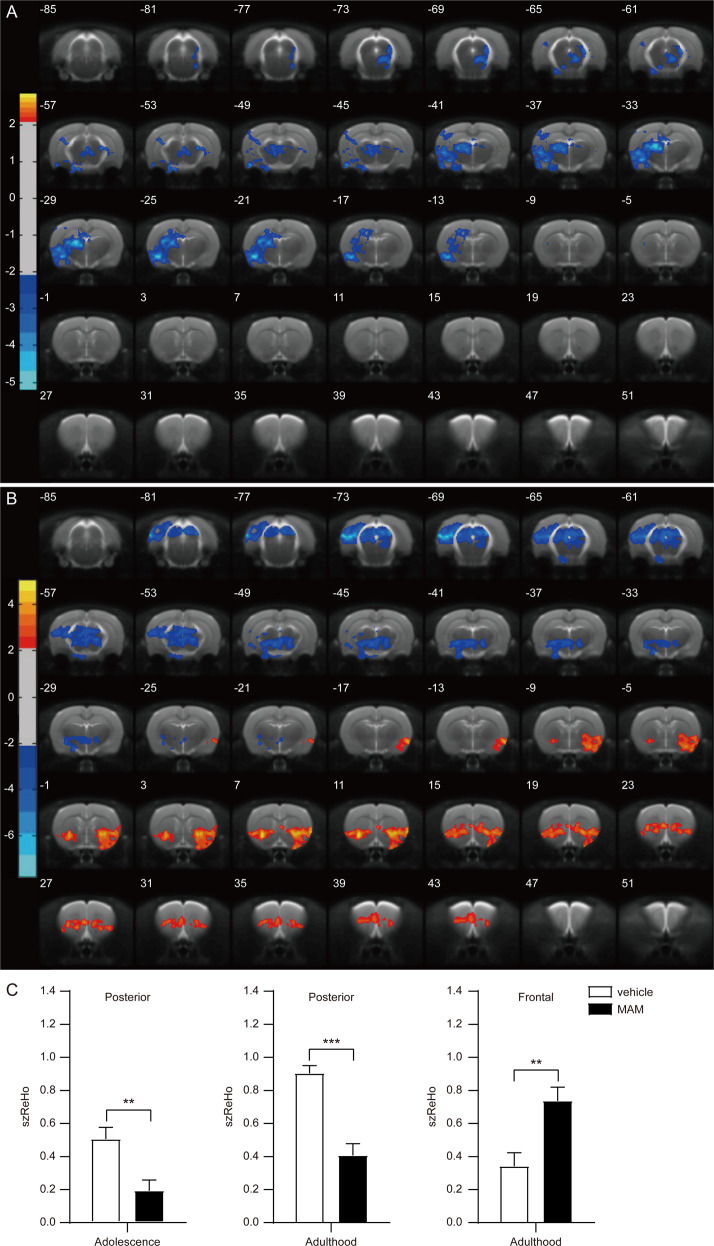
Aberrant function developmental patterns in MAM rats. **A** In adolescence, MAM rats showed significantly decreased ReHo in the posterior region (visual and associated cortices). **B** In adulthood, MAM rats showed significantly increased ReHo in the frontal region (frontal cortices) and decreased ReHo in the posterior region (visual and associated cortices). **C** ReHo value was extracted from the overlaps between the frontal and posterior ROIs and the clusters identified in voxel-based tests of animal longitudinal study. In adolescence, ReHo value of MAM rats was significantly decreased in the posterior overlap, compared to vehicle rats. In adulthood, ReHo value of MAM rats was significantly increased in the frontal overlap and significantly decreased in the posterior overlap, compared to vehicle rats. **A**, **B** Significant at voxel *P* value < 0.05, cluster *P* value < 0.05 corrected by GRF correction; **C** significant at *P* < 0.05, *P < 0.05, **P < 0.01, ***P < 0.001; the frontal ROI contains three subareas: the medial prefrontal cortex, orbitofrontal cortex, and anterior cingulate cortex; the posterior ROI contains three subareas: visual cortex, auditory cortex, and temporal association cortex; posterior, the overlaps between the posterior ROI and the clusters in the posterior region identified in voxel-based tests of animal longitudinal study; frontal, the overlap between the frontal ROI and the cluster in the frontal region identified in a voxel-based test of an animal longitudinal study. MAM methylazoxymethanol acetate, ROI region of interest.

In adolescence, MAM rats had significantly decreased ReHo in the posterior regions, including left auditory cortex, left temporal association cortex, left visual cortex, left entorhinal cortex, left somatosensory cortex, bilateral amygdala, and bilateral hippocampus, compared to vehicle rats (Fig. 3A) (Supplementary Table 3). In adulthood, MAM rats had significantly increased ReHo in frontal regions, including bilateral medial prefrontal cortices (mPFC), bilateral OFC, bilateral nucleus accumbens, and bilateral striatum, and significantly decreased ReHo in posterior regions, including left visual cortex, left auditory cortex, left temporal association cortex, and bilateral hippocampus, compared to vehicle rats (Fig. 3B) (Supplementary Table 4).

Two ROIs were defined based on results from ReHo analyses in humans and rats: (1) The frontal ROI included three subareas: mPFC, OFC, and ACC, and (2) the posterior ROI included three subareas: visual cortex, auditory cortex, and temporal association cortices. For post hoc comparisons, ReHo value was extracted from the overlaps between the frontal and posterior ROIs and the significant clusters identified in the longitudinal analysis of MAM rats. Compared to vehicle rats, MAM rats had significantly decreased ReHo value within the posterior overlap in adolescence (*T* = −3.241, *P* = 0.004) (Fig. 3C). In adulthood, ReHo value was significantly increased within the frontal overlap (T = 3.364, *P* = 0.004) and significantly decreased within the posterior overlap in MAM rats (*T* = −5.694, *P* < 0.001), compared to vehicle rats (Fig. 3C). MAM rats demonstrated aberrant developmental evolution in brain function from primarily decreased posterior ReHo in adolescence to further decreased posterior ReHo along with increased frontal ReHo in adulthood.

The findings supported that shared frontal-posterior functional imbalance between hSZ and adult MAM rats. The longitudinal ReHo changes from adolescence to adulthood in MAM rats may simulate neurodevelopmental patterns in hSZ. Moreover, the visual cortex was a common brain region of ReHo alterations in hSZ and adolescent MAM rats and adult MAM rats, and ReHo alterations in posterior regions including the visual cortex in adolescence appeared to precede further progression in a frontal-posterior functional imbalance in adulthood. Altogether, it supported that the visual cortex was a critical locus and adolescence was a sensitive window in SZ development, which has the neuromodulatory potential for frontal-posterior functional imbalance in MAM rats.

### Potential mechanisms of frontal-posterior functional imbalance in MAM rats

**Fig. 4:**
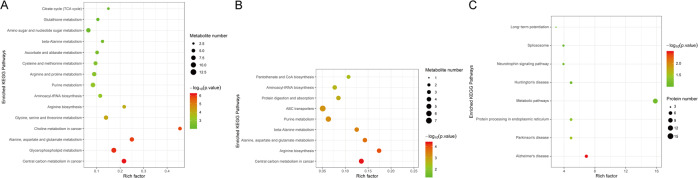
Pathway characteristics of frontal-posterior functional imbalance in MAM rats. **A** Pathways of metabolomic profiles in the PFC between vehicle rats and MAM rats. The metabolites are enriched in the central carbon metabolism pathway. **B** Pathways of metabolomic profiles in the OC between vehicle rats and MAM rats. The metabolites are enriched in the central carbon metabolism pathway. **C** Pathways of proteomic profiles in the prefrontal cortex between vehicle-sham rats and MAM-sham rats. The significant network was the “Alzheimer disease pathway”. MAM methylazoxymethanol acetate, PFC prefrontal cortex, OC occipital cortex, KEGG Kyoto Encyclopedia of Genes and Genomes.

In the brain tissue metabolomic analysis, a total of 44,766 metabolic features were detected, including 26,045 in positive ion mode and 18,721 in negative ion mode. The statistical analysis of internal standard peak intensity of all samples showed sufficient repeatability (relative standard deviation < 30%), permitting further analysis. Significant differences between MAM rats and vehicle rats were found in 66 metabolites in the PFC and 90 metabolites in the occipital cortex (OC). The KEGG pathway detected “Central carbon metabolism disturbance” both in the PFC and OC of MAM rats (Fig. 4A, B). Moreover, in the PFC, l-Glutamate, l-Glutamine, d-Fructose 1,6-bisphosphate, l-Alanine, l-Aspartate, l-Malic acid, phosphoenolpyruvate, and l-Histidine were disturbed, covering glycolysis and gluconeogenesis, citrate cycle, alanine, aspartate and glutamate metabolism, glycine, serine and threonine metabolism, arginine biosynthesis, glycerophospholipid metabolism, and pentose phosphate pathways (Supplementary Table 5). In the OC, l-Glutamine, l-Valine, l-Aspartate, Citrate, and l-Histidine were disturbed, covering citrate cycle, alanine, aspartate, and glutamate metabolism and pentose phosphate pathways (Supplementary Table 5). These results suggest disturbances in central carbon metabolisms as potential mechanisms for frontal-posterior functional imbalance in MAM rats.

In brain tissue proteomic analysis, 165 proteins in the PFC and 27 proteins in the OC showed differential expression between MAM rats and vehicle rats. When comparing MAM rats with vehicle rats, the altered proteins of the PFC were enriched in the “Alzheimer disease pathway” (Fig. 4C), containing proteins related to mitochondrial dysfunction (Supplementary Fig. 4). There was no significant pathway in the OC. These results suggest that altered mitochondrial function in the PFC may contribute to frontal-posterior functional imbalance in MAM rats.

## Discussion

In this study, we found a similar frontal-posterior functional imbalance between hSZ and MAM rats, characterized as increased frontal ReHo (frontal cortices), and decreased posterior ReHo (visual and associated cortices). Longitudinal analysis of brain function changes in MAM rats revealed that aberrant functional patterns progressed from adolescence to adulthood: primarily decreased posterior ReHo (visual and associated cortices) to further decreases in posterior ReHo and increased frontal ReHo. Accordingly, it supported that the visual cortex was a critical locus and adolescence was a sensitive window in SZ development, which has the neuromodulatory potential for frontal-posterior functional imbalance in MAM rats. In addition, metabolic and proteomic changes suggested the potential mechanisms underlying ReHo alterations in adulthood, including central carbon metabolism disturbance and mitochondrial dysfunction. The understanding of the dynamic nature underlying SZ is crucial for the development of effective therapies, which have significant translational implications for SZ.

Frontal-posterior functional imbalance consisting of significantly increased ReHo in higher-order frontal cortices and significantly decreased ReHo in the posterior primary sensory cortices was observed in both hSZ and MAM rats. Our previous studies have shown a similar functional imbalance between frontal and posterior regions with significantly increased ALFF and FC in the prefrontal and orbitofrontal cortices, and significantly decreased ALFF and FC in the primary sensory cortices in SZ [20, 33]. We have also found disrupted balance in brain network segregation and integration in SZ with significantly decreased short-range connectivity in the primary sensory cortices and significantly increased medium/long-range connectivity in the PFC [21]. A recent meta-analysis study examining spatial and temporal intrinsic brain activity in SZ found that alterations in ALFF/fALFF and ReHo have a similar distribution in SZ, such as decreased ALFF/fALFF and ReHo in the somatosensory, posterior parietal, and occipital cortices, and increased ALFF/fALFF and ReHo in the striatum, medial temporal and prefrontal cortices [34].

Furthermore, we found that MAM rats showed decreased ReHo in the primary sensory cortices in adolescence. These findings in adolescent MAM rats may relate to functional brain alterations in clinical high-risk groups for SZ (CHR-SZ) or first-degree relatives of hSZ. Studies have shown significantly reduced ReHo in the right insula and right superior temporal gyrus [35], as well as left inferior occipital gyrus [36], in first-degree relatives of SZ individuals, compared to HC. Similarly, functional brain changes with significantly decreased ALFF and FC in the posterior sensory cortices were also found. CHR-SZ has shown decreased FC of precuneus and visual association areas [37], relative to HC. Moreover, significantly decreased ALFF in the left inferior parietal lobule and postcentral gyrus have been found in CHR-SZ compared to HC [38].

As SZ is a neurodevelopmental disorder, a major focus has been to understand what brain changes and what regions, and which time initiate the brain functional alterations that lead to SZ. In this study, our results supported that the adolescent visual cortex was a critical locus and a sensitive window in SZ development based on the longitudinal data of brain function changes from adolescence to adulthood in MAM rats. Adolescence is considered as a time-sensitive window of brain development, during which robust brain plasticity is exhibited [39]. In adolescence, the chronological desynchronization of spontaneous neuronal activity corresponds to the increased sensory input signals, switching the factors most strongly to drive the shaping and development of cortical networks [40]. Any changes in synaptic plasticity during adolescence have long-lasting effects on brain function [41]. Moreover, primary sensory cortices appear to have essential roles in brain development. The development of primary sensory regions, particularly regions concerning visual and auditory streams, precedes and scaffolds higher-order regions structure and function, like the PFC [42]. The dysfunction of primary sensory regions could result in aberrant processing and translation of afferent sensory inputs and a cascade of distinct events in the higher-order regions, and thus inaccurate integration of sensory signals and erroneous feedback within sensory processing circuits lead to progressive and persistent disruptions within the brain function in SZ [43, 44].

The potential mechanisms underlying frontal-posterior functional imbalance are of significant interest as they could provide a greater understanding of the biological changes of functional alterations and facilitate treatment development for SZ. Central carbon metabolism disturbance was detected in MAM rats. This pathway mainly refers to the altered glucose metabolism, which plays an essential role in the supply of biosynthetic precursors and energy [45]. In our study, energy metabolism appeared altered in the PFC and OC in MAM rats. The frontal-posterior functional imbalance, induced by disturbed cerebral blood flow, may indicate abnormal energy demand in response to abnormally high glucose demand or insufficient glucose supply in regional cortices [46]. The consumption of glucose resulted in an increase of lactate levels [47], which was associated with oxidative stress, dopamine reuptake inhibition, synaptic glutamate release, and cognitive impairment, which is in line with SZ features [48–50]. Proteomic analyses implicated mitochondrial dysfunction in MAM rats that could disrupt the bioenergetics in the developing brain and lead to neurodevelopmental abnormalities [51]. Mitochondrial function can disturb calcium homeostasis, which changes the spontaneous neocortical activity and resting-state fMRI [52, 53]. Reduced or enhanced mitochondrial calcium uptake capacity would decrease or increase spontaneous neuronal activity, respectively [54]. Moreover, altered mitochondrial calcium homeostasis could lead to dopamine and glutamate system dysfunctions, and thus ultimately affect synaptic plasticity and cortical microcircuitry in SZ [55].

Several limitations should be noted. First, the anesthetic agents used for MRI scanning may change neuronal functions and neurovascular coupling. The potential confounding effects due to anesthesia should have been minimized with comparison to controls that were also anesthetized for scanning. Second, we do not provide information about cellular changes involved in the dynamic maturational processes of MAM rats, and therefore further assessment of cellular changes from adolescence to adulthood should be supplemented for comprehensive phenotyping. Third, although MAM rats are a validated animal model of SZ, no single animal model can fully capture the complex neurobiological and behavioral spectrum of SZ. Future studies using additional preclinical models are needed for a more comprehensive understanding of SZ.

In summary, based on our findings in hSZ and the neurodevelopmental MAM rat model of SZ, the frontal-posterior functional imbalance appears to be a significant feature in SZ, and likely frontal-posterior functional imbalance undergoes aberrant development from adolescent decreased posterior brain function (visual and associated cortices) to adult increased frontal (frontal cortices)-decreased posterior brain function. Further, metabolic and proteomic changes in MAM rats suggest central carbon metabolism disturbance and mitochondrial dysfunction as potential mechanisms of frontal-posterior functional imbalance in adult SZ. These findings link data between humans and animal models in SZ and may have a significant translational contribution to effective neuromodulation in SZ. This work is the first of its kind and will need further study and replication.

## Supplementary information

Supplementary data

## Data Availability

The metabolic and proteomic data, the fMRI datasets generated and analyzed in this study are available upon request.

## Code availability

We also freely provide the code used to generate the data.
