# Supplementary material for: Frontal-posterior functional imbalance and aberrant function developmental patterns in schizophrenia
Source: Transl Psychiatry. 2021 Sep 27;11:495. doi: 10.1038/s41398-021-01617-y (PMC8476507; doi:10.1038/s41398-021-01617-y)
Supplement: Supplementary file 1 — Supplementary data [file 41398_2021_1617_MOESM1_ESM.doc]

**Supplemental data**

**Including Contents**

**Supplemental Methods**

Animal behavioral assessment

Animal metabolomics profiling

Animal proteomics profiling

Statistical analysis

**Supplemental Results**

Human fMRI supplementary results

Animal behavioral supplementary results

Animal fMRI supplementary results

Potential mechanisms supplementary results

**Supplemental Figures**

Supplementary Figure 1 Behavioral differences between vehicle rats and MAM rats in adolescence and adulthood

Supplementary Figure 2 The escape latency in the acquisition trials of MWM between vehicle rats and MAM rats in adolescence and adulthood

Supplementary Figure 3 Correlations between mean ReHo value in the posterior overlaps and the behavioral variables of MAM rats in adolescence and adulthood

Supplementary Figure 4 Alzheimer disease networks of proteomic analysis in the PFC between vehicle rats and MAM rats.

**Supplemental Tables**

Supplementary Table 1 Demographic and clinical characteristics of HC and hSZ

Supplementary Table 2 ReHo differences in brain regions between HC and hSZ

Supplementary Table 3 ReHo differences in brain regions between vehicle rats and MAM rats in adolescence

Supplementary Table 4 ReHo differences in brain regions between vehicle rats and MAM rats in adulthood

Supplementary Table 5 Metabolites that enriched in central carbon metabolism in the PFC and the OC

**References**

**Supplemental Methods**

**Animal behavioral assessment**

The open field test (OFT) was conducted in the apparatus with a square, black box (100×100×100cm^3^) and a video camera above. The central 10×10cm^2^ area was defined as the center zone. Each animal was placed in the center, and allowed to explore freely in the open field. The travel path was tracked by the video camera for 5 min. The box was cleaned with 10% ethanol after each test. The total distance traveled (distance), entries to the center area (center entries), and time spent in the center area (center time) were analyzed using video-tracking software (ANY-maze, Stoelting CO, USA)[^1^](#_ENREF_1).

The Morris water maze (MWM) was conducted in a circular black pool with a diameter of 180cm and a height of 50cm, filled with opaque water at 24±1.0°C, and a video camera above. The pool was divided into four equivalent quadrants, i.e. northeast (NE), northwest (NW), southeast (SE), and southwest (SW), with visible external cue on each quadrant wall. An escape platform (10 cm diameter) was submerged 2 cm underwater in the center of SW quadrant. The duration of the experiment was six days. On day 0 of adaptation, the rats were placed individually into the pool for 60 sec. On day 1 to 4 of acquisition, four trials were performed in each day, starting on NE quadrant, followed by NW, SE and SW quadrant in turn. For each trial, the rats were individually placed in the water facing the pool wall and allowed to swim freely until they found the platform and stayed on it for 3 sec. If one failed to find the platform within 60 sec, it was guided to the platform and stayed for 10 sec. On day 5 of probe trial, each animal was placed in the pool without the platform, and allowed to swim freely for 90 sec. Time to the platform (escape latency) in each acquisition trial and probe trial was calculated. Time spent in the target quadrant (quadrant time) and the number of times the subject crossed the platform location (platform crossing) were calculated during the probe trial. The ANY-maze software was utilized to record and analyze this experiment[^2^](#_ENREF_2).

**Animal metabolomics profiling**

Rats were sacrificed by rapid decapitation after isoflurane exposure on postnatal day (PD) 73. The brains were obtained and rinsed in ice-cold DEPC-treated water. With the guide of brain maps 4.0[^3^](#_ENREF_3), the targeted cortices (prefrontal and occipital cortices) were located and dissected quickly, snap-frozen in liquid nitrogen and then stored at -80°C until analysis. The frozen brain sample (60mg) was thawed at 4°C, and vortexed in 1ml of cold methanol/acetonitrile/H2O (2:2:1, v/v/v), following by homogenizing with a homogenizer (FastPrep-24™, MP Biomedicals LLC., Santa Ana, California, USA) at 6.0M/S (60s each, twice). The homogenate was sonicated at low temperature (30min/once, twice). Then, the mixture was centrifuged for 20 min (14000g, 4°C), and the supernatant was dried in a vacuum centrifuge and stored at −80°C until redissolution in 100μl of an acetonitrile/water (1:1, v/v) solvent for metabolomic analysis.

The untargeted metabolomic analysis was performed using an ultra-performance liquid chromatography (UPLC) system (1290 Infinity LC, Agilent Technologies, Santa Clara, California, USA) coupled to a quadrupole time-of-flight (TOF) mass spectrometer (Triple TOF 6600, AB Sciex, Framingham, MA, USA) with electrospray ionization (ESI) in a positive and negative ionization modes. Samples were separated using a 2.1mm × 100mm ACQUIY UPLC BEH 1.7µm column (Waters, Ireland) with a column temperature of 25°C. In both ESI positive and negative modes, the mobile phase contained A=25mM ammonium acetate and 25mM ammonium hydroxide in water and B = acetonitrile. The gradient was 85% B for 1min, was a linearly reduced to 65% in 11min, and then was reduced to 40% in 0.1min and kept for 4min, and then increased to 85% in 0.1min, with a 5min re-equilibration period. In mass spectrometric (MS) analysis, the ESI source conditions were set as follows: ion source gas 1 (Gas1) of 60 psi, ion source gas 2 (Gas2) of 60 psi, curtain gas (CUR) of 30 psi, source temperature of 600°C, and ion spray voltage floating of ±5500V. For the MS only acquisition, the instrument was set to acquire data over the m/z range of 60–1000 Da, and the accumulation time for TOF MS scan was set at 0.20 s/spectrum. For auto MS/MS acquisition, the instrument was set to acquire over the m/z range 25-1000 Da, and the accumulation time for product ion scan was set at 0.05 s/spectrum. The product ion scan was operated using information dependent acquisition (IDA) with high sensitivity mode. The parameters were as follows: collision energy (CE), 35 V with ±15 eV; declustering potential (DP), 60V (+) and −60V (−); exclude isotopes within 4Da; candidate ions to monitor per cycle: 10.

The raw MS data (wiff.scan files) were converted to MzXML files using ProteoWizard MSConvert before importing into freely available XCMS software. In peak picking, the parameters were as follows: centWave m/z=25ppm, peakwidth=c (10, 60), and prefilter=c (10, 100). In peak grouping, the following parameters were used: bw=5, mzwid=0.025, minfrac=0.5. Collection of Algorithms of Metabolite Profile Annotation (CAMERA) was used for annotation of isotopes and adducts. And only the variables greater than 50% of the nonzero measurement values in at least one group were kept. Compound identification of metabolites was performed by comparing the accuracy m/z value (<25ppm), and MS/MS spectra with a self-built database established with available authentic standards. After normalized to total peak intensity, the processed data were uploaded into MetaboAnalyst software for further analysis (www.metaboanalyst.ca).

**Animal proteomics profiling.**

Brain sample collection was in the same way as mentioned above in metabolomic profiling. The frozen brain sample (60mg) was thawed at 4°C, resuspended in 100μl SDT lysis buffer (4%SDS, 100mM Tris-HCl, 1mM DTT, pH7.6), and homogenized by MP homogenizer (24×2, 6.0M/S, 60s, twice). The homogenate was sonicated for 1 min, boiled for 15 min, and then centrifuged at 14000g for 40 min. The supernatant was filtered with filters (0.22µm), and quantified with BCA Protein Assay Kit (Bio-Rad, USA). Protein of each sample (200μg) was incorporated into 30μl SDT buffer (4% SDS, 100mM DTT, 150mM Tris-HCl, pH 8.0). UA buffer (8M Urea, 150mM Tris-HCl, pH 8.0) was added to remove DTT and other low-molecular-weight components by repeated ultrafiltration (Microcon units, 10kD). And 100μl iodoacetamide (100mM IAA in UA buffer) was added to block reduced cysteine residues. After incubation in darkness for 30min, the sample was washed with 100μl UA buffer three times and 100μl 25mM NH4HCO3 buffer twice. The suspension was digested with 4μg trypsin (Promega) in 40μl 25mM NH4HCO3 buffer overnight at 37°C. Finally, the peptide was desalted on C18 Cartridges (Empore™ SPE Cartridges C18, bed I.D. 7mm, volume 3ml, Sigma), concentrated by vacuum centrifugation, and reconstituted in 40µl 0.1% (v/v) formic acid for LC-MS/MS analysis.

Samples were performed on a Q Exactive mass spectrometer that was coupled to Easy nanoLC (Thermo Fisher Scientific). Samples were loaded onto a reverse phase trap column (Thermo Scientific Acclaim PepMap100, 100μm*2cm, nanoViper C18) connected to the C18-reversed phase analytical column (Thermo Scientific Easy Column, 10cm long, 75μm inner diameter, 3μm resin) in buffer A (0.1% formic acid) and separated with a linear gradient of buffer B (84% acetonitrile and 0.1% formic acid) at a flow rate of 300 nl/min controlled by intelliflow technology. A 2 hours gradient was performed with 0-55% buffer B for 110min, 55-100% buffer B for 5min, and hold in 100% buffer B for 5min. The MS analysis was operated in positive ion mode. MS data was acquired using a data-dependent top10 method dynamically choosing the most abundant precursor ions from the survey scan (300–1800m/z) for HCD fragmentation. Automatic gain control (AGC) target was set to 3e6, and maximum inject time to 10ms. Dynamic exclusion duration was 25s. Survey scans were acquired at a resolution of 70000 at m/z 200 and resolution for HCD spectra was set to 17500 at m/z 200. Normalized collision energy was 30eV, and the underfill ratio was defined as 0.1%. The instrument was run with peptide recognition mode enabled.

The MS data were analyzed using MaxQuant software version 1.3.0.5 (Max Planck Institute of Biochemistry in Martinsried, Germany). The search parameters were as following: Enzyme: trypsin; Max missed cleavages: two; Main search: 6ppm; First search: 20pm; MS/MS tolerance: 20 ppm; Fixed modifications: carbamidomethyl (C); Variable modifications: oxidation (M) and acetyl (Protein N-term); Database: Ensembl_Rattus_29107_20190628.fasta, uniprot_mouse_76417_20141212.fasta, and a self-built database created using authentic standards; Database pattern: reverse. The cutoff of global false discovery rate (FDR) for peptide and protein identification was set to 0.01. Label-free quantification was carried out in MaxQuant. Protein aboundance was calculated on the basis of the normalized spectral protein intensity (LFQ intensity). The MS results were forwarded to statistical analysis.

**Statistical analysis**

For animal behavioral data, the indexes of distance, center entries and center time were analyzed using independent two-sample t-tests in the OFT. For analysis of the acquisition trials of MWM, escape latency from the four trials in each training day was calculated in average. Escape latency was analyzed using two-way repeated measures (RM) analysis of variance (ANOVA) with days as RM. The independent two-sample t-test was used to further test the difference between vehicle and MAM rats on each training day. For analysis of the probe trial of MWM, the indexes of escape latency, platform crossings and quadrant time were analyzed by independent two-sample t-tests. The criterion for statistical significance was a probability value of 0.05.

We also investigated the potential relationships of mean ReHo values with clinical characteristics in hSZ and with the behavior variables in MAM rats. In hSZ, partial correlations controlling for age and sex were performed between mean ReHo values and illness duration, BPRS scores or WCST scores. In MAM rats, pearson bivariate correlations were performed between mean ReHo values and the behavior variables, including distance, center entries, center time in the OFT, and escape latency, platform crossing, quadrant time in the probe trial of MWM. Statistical significance was set at *P*<0.05.

**Supplemental results**

**Human fMRI cross-sectional studies.**

The demographic information and clinical features were presented in Supplemental Table 1. There was no significant difference in age, sex, or handedness between hSZ and HC (*P*>0.05). Significant differences were observed in three categories: educational level (*P*<0.001), BPRS total and subscale scores (*P*<0.001), and WCST scores (*P*=0.001 in corrected responses, *P*=0.002 in categories completed, *P*=0.001 in total errors, *P*=0.025 in perseverative errors and *P*=0.011 in nonperseverative errors, respectively) between hSZ and HC.

Mean ReHo values in the heteromodal, limbic, paralimbic cortical and subcortical regions (Cluster 1) were positively correlated with BPRS total score (r=0.527, *P*=0.001), BPRS subscale score (lack of energy) (r=0.584, *P*<0.001), and nonperseverative errors (r=0.505, *P*=0.032) in hSZ. Mean ReHo values in another heteromodal, limbic and paralimbic cortical regions (Cluster 2) were also positively correlated with BPRS total score (r=0.338, *P*=0.047), BPRS subscale score, including lack of energy (r=0.390, *P*=0.021) and thought disorder (r=0.361, *P*=0.033), and the nonperseverative errors (r=0.685, *P*=0.002) in hSZ. Furthermore, mean ReHo values in the primary sensory cortical regions (Cluster 6) were negatively correlated with illness duration (r=-0.351, *P*=0.036; r=-0.437, *P*=0.008).

**Animal longitudinal studies.**

In adolescence, fMRI data of 12 MAM rats and 11 vehicle rats were available, OFT data of 12 MAM rats and ten vehicle rats were available, and MWM data of 12 MAM rats and 11 vehicle rats were available. In adulthood, fMRI data of ten MAM rats and nine vehicle rats were available, OFT data of 11 MAM rats and ten vehicle rats were available, MWM data of 11 MAM rats and ten vehicle rats were available.

In adolescence, MAM rats exhibited anxiety-like behavior in the OFT, manifesting as less time spent in the center area (T=-3.273, *P*=0.004) and fewer entries to the center area (T=-2.322, *P*=0.031), compared to vehicle rats (Supplemental Figure 1A, B). There was no difference in total travel distance between MAM and vehicle rats (*P*>0.05) (Supplemental Figure 1C). For cognitive analysis, there was no group effect on any index of MWM (*P*>0.05) (Supplemental Figure 1D-F and Supplemental Figure 2A). A significant positive correlation (r=0.587, *P*=0.045) was found between mean ReHo value in the posterior overlap and center time in the OFT (Supplemental Figure 3A), which indicated that as mean ReHo value in the posterior overlap decreased, MAM rats spent less time in center area, exhibiting increased anxiety-like behavior. There was no significant correlation between mean ReHo value in the posterior overlap and distance or center entries in the OFT, nor escape latency, quadrant time, or platform crossing in the probe trial of MWM (*P*>0.05).

In adulthood, MAM rats showed impairment in spontaneous locomotor activity, anxiety-like behavior state, cognitive deficits in reference memory, and impairment in spatial learning ability. Specifically, in the OFT, compared to vehicle rats, MAM rats exhibited shorter total travel distance (T=-2.776, *P*=0.012), and fewer entries to the center area (T=-2.27, *P*=0.035) (Supplemental Figure 1B, C). However, there was no significant difference in time spent in the center area (*P*>0.05) (Supplemental Figure 1A). In comparison with vehicle rats, MAM rats spent more quadrant time in the probe trial of MWM (T=2.707, *P*=0.014) (Supplemental Figure 1D). There was no group effect on any other index of the probe trial (*P*>0.05) (Supplemental Figure 1E, F). The two-way ANOVA with day as repeated measures revealed a group effect on escape latency in the acquisition trials of MWM (F=8.603, *P*=0.008). Further independent two-sample t-test revealed that MAM rats spent more time to find the platform on day 2 (T=2.632, *P*=0.016) and day 4 (F=3.894, *P*=0.001) in the acquisition trials of MWM (Supplemental Figure 2B). Correlation analysis showed a significant negative correlation (r=-0.673, *P*=0.033) between mean ReHo value in the posterior overlap and escape latency in the probe trial of MWM (Supplemental Figure 3B), which indicated that as mean ReHo value in the posterior overlap decreased, MAM rats spent more time trying to find the platform, showing a cognitive deficit in reference memory. There was no significant correlation between mean ReHo value in the posterior overlap and quadrant time, or platform crossing in the probe trial of MWM, nor distance, center entries or center time in the OFT. We found no significant correlation between mean ReHo value in the anterior overlap and any behavioral variable.

Collectively, both hSZ and adult MAM rats showed the common behavioral changes, expressing as anxiety status and cognitive deficits.

**Supplementary Figures**


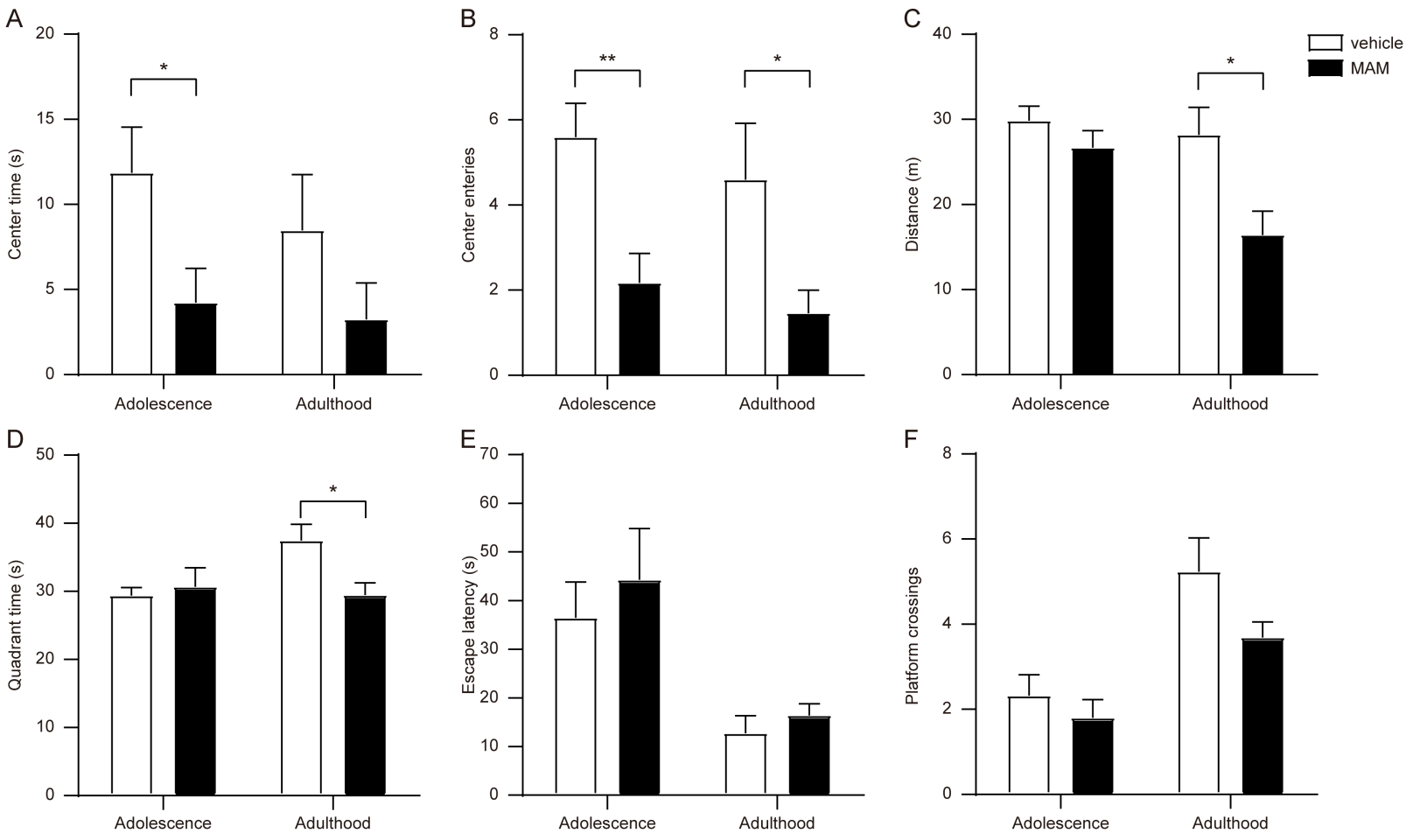


**Supplementary Figure 1. Behavioral differences between vehicle rats and MAM rats in adolescence and adulthood.**

(A), (B) and (C) In OFT, MAM rats showed anxiety-like behavior state in both adolescence and adulthood, and impairment in spontaneous locomotor activity in adulthood. (A) MAM rats spent less time in the center area than vehicle rats in adolescence. (B) MAM rats showed fewer entries to the center area than vehicle rats in both adolescence and adulthood. (C) MAM rats traveled less distance than vehicle rats in adulthood. (D), (E) and (F) In MWM, MAM rats showed impairment in spatial learning ability in adulthood. (D) MAM rats spent less quadrant time in probe trial than vehicle rats. (E) and (F) There was no group effect on escape latency and platform crossing in probe trial in both adolescence and adulthood. (A)-(F) Data were presented as mean±SEM. Significant at *P*<0.05, ******P*<0.05, *******P*<0.01, ********P*<0.001.


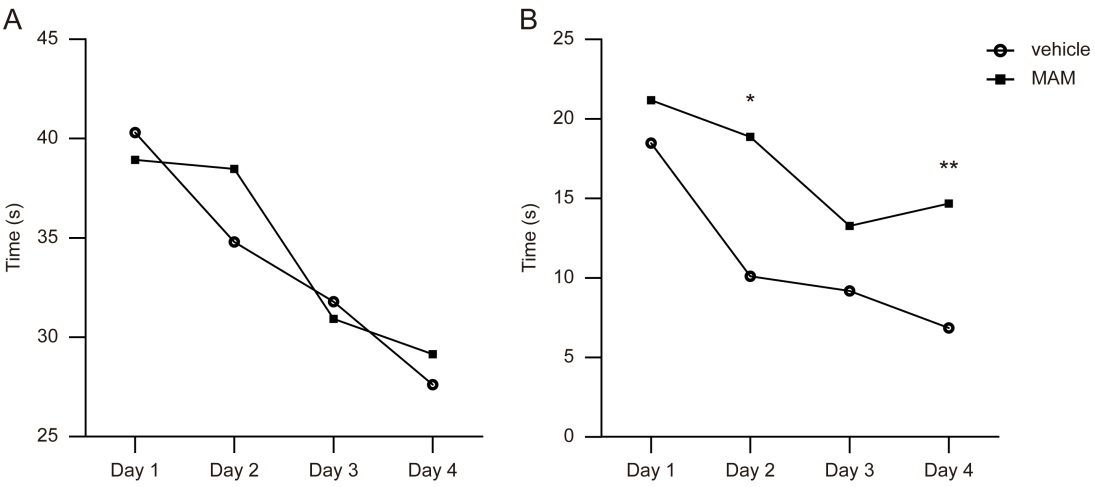


**Supplementary Figure 2. The escape latency in the acquisition trials of MWM between vehicle rats and MAM rats in adolescence and adulthood.**

(A) In MWM, there was no group effect on escape latency in the acquisition trials in adolescence. (B) In MWM, there was a group effect on escape latency in the acquisition trials in adulthood. MAM rats spent more time to find the platform on day 2 and 4. (A) and (B) Data were presented as mean. Significant at *P*<0.05, ******P*<0.05, *******P*<0.01, ********P*<0.001.

**
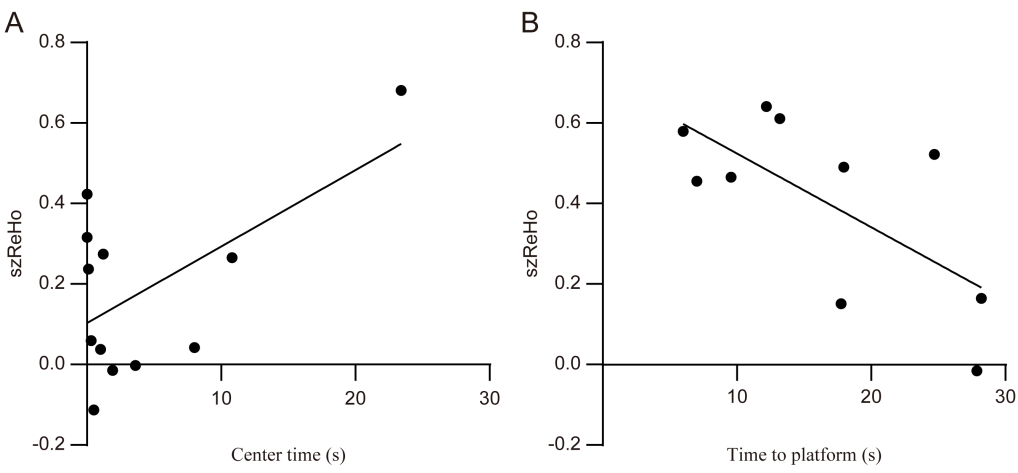
**

**Supplementary Figure 3. Correlations between mean ReHo value in the posterior overlaps and the behavioral variables of MAM rats in adolescence and adulthood.**

(A) There was a positive correlation between mean ReHo value of the posterior overlap and center time of the OFT in adolescence. (B) There was a negative correlation between mean ReHo value of the posterior overlap and escape latency in the probe trial of MWM in adulthood. The posterior ROI contains three subareas: visual cortex, auditory cortex, and temporal association cortex. Posterior overlaps, the overlaps between the posterior ROI and the clusters in the posterior region identified in voxel-based tests of animal longitudinal study.

**
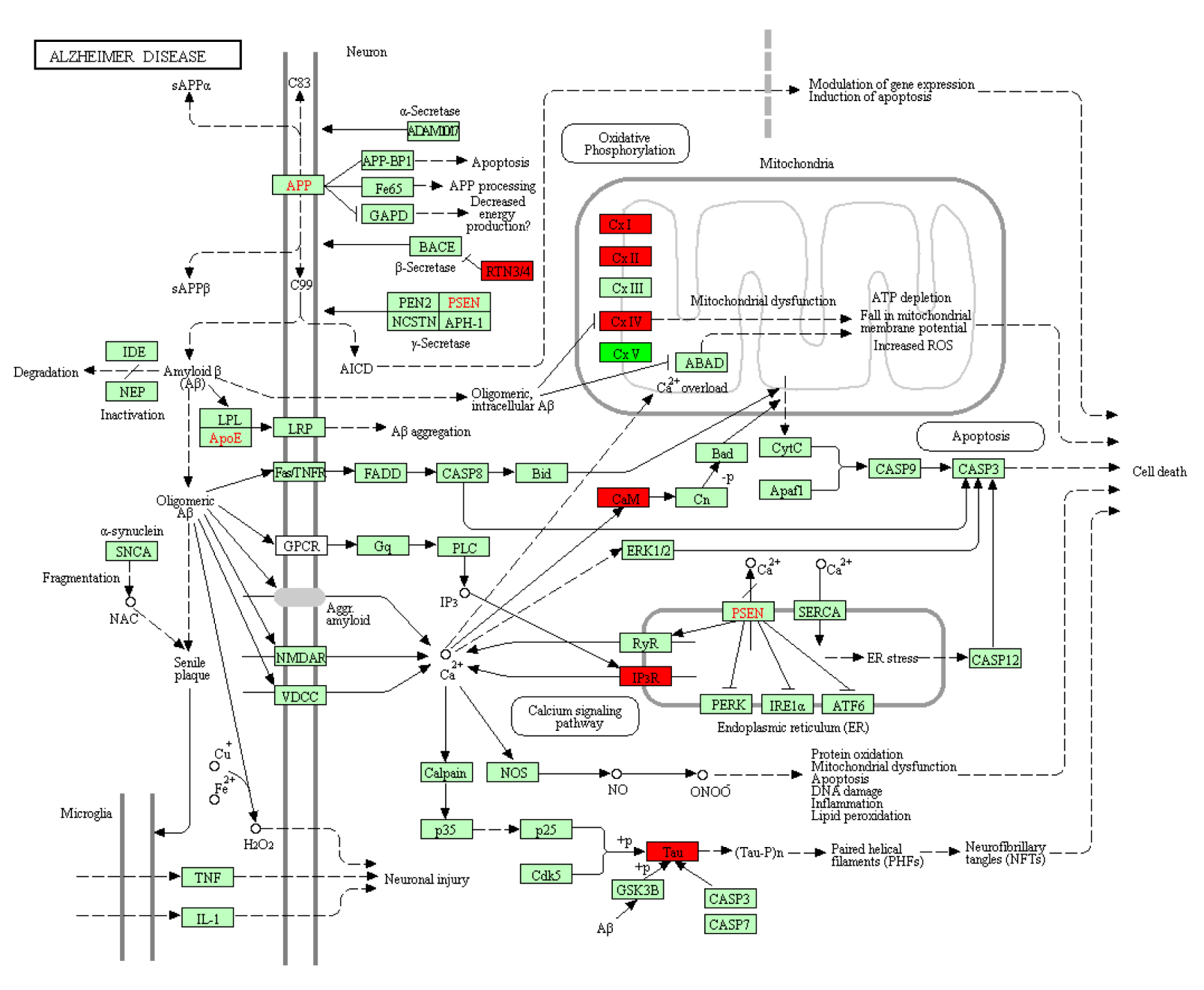
**

**Supplementary Figure 4.** **Alzheimer disease networks of proteomic analysis in the PFC between vehicle rats and MAM rats.**

The ‘Alzheimer disease pathway’ enriched with proteins related to mitochondrial dysfunction, when MAM-sham rats compared to vehicle-sham rats. Red color represents upregulated proteins; Dark green color represents downregulated proteins; Light green color represents no significance.

**Supplementary Tables**

**Supplementary Table 1. Demographic and clinical characteristics of HC and hSZ in human and animal fMRI cross-validation design.**

Data are presented as n (%) or mean±SD.

| Variables | HC | hSZ | T/χ^2^ | *P* |
| --- | --- | --- | --- | --- |
|  | (n=42) | (n=40) |  |  |
| Age (year) | 29.17±7.97 | 28.15±7.96 | -0.578 | 0.565 |
| Sex (%) | 38.10 | 40.00 | 0.031 | 0.860 |
| Education (years) | 14.34±3.34 | 10.75±3.05 | -5.045 | <0.001 |
| Right-handed (%) | 88.10 | 90.00 | 0.076 | 0.783 |
| Duration (month) | ‒ | 7.86±11.42 | ‒ | ‒ |
| Total BPRS | 18.55±1.48 | 43.19±13.45 | 10.46 | <0.001 |
| Anxiety and depression | 4.36±0.93 | 9.54±4.13 | 7.032 | <0.001 |
| Lack of energy | 4.03±0.17 | 9.05±4.41 | 6.535 | <0.001 |
| Thought disorder | 4±0 | 10.65±4.28 | 8.91 | <0.001 |
| Activity | 3.15±0.51 | 5.00±2.17 | 4.768 | <0.001 |
| Hostility | 3.00±0 | 8.86±4.14 | 8.123 | <0.001 |
| WCST |  |  |  |  |
| Corrected responses | 32.25±10.33 | 21.00±12.11 | -3.575 | 0.001 |
| Categories completed | 3.94±2.15 | 1.95±2.01 | -3.318 | 0.002 |
| Total errors | 15.75±10.33 | 27.00±12.11 | 3.575 | 0.001 |
| Perseverative errors | 4.88±5.08 | 10.40±11.97 | 2.309 | 0.025 |
| Nonperseverative errors | 10.91±6.97 | 16.60±8.38 | 2.652 | 0.011 |

**Supplementary Table 2. ReHo differences in brain regions between HC and hSZ.**

| Brain region | Brodmann area | Cluster size | Peak MNI coordinates | | | T***** |
| --- | --- | --- | --- | --- | --- | --- |
|  |  |  | X | Y | Z |  |
| Cluster 1 |  |  |  |  |  |  |
| Bilateral posterior cingulate gyrus, bilateral median cingulate and paracingulate gyri, left hippocampus, left parahippocampal gyrus, left fusiform gyrus, left inferior temporal gyrus, bilateral precuneus | 31, 23, 24, 27, 34, 28, 37, 20, 7 | 833 | -48 | -15 | -33 | 5.430 |
| Cluster 2 |  |  |  |  |  |  |
| Bilateral superior frontal gyrus, bilateral inferior frontal gyrus, bilateral middle frontal gyrus, bilateral anterior cingulate and paracingulate gyri, bilateral insula, bilateral rectus gyrus, bilateral temporal pole, right fusiform gyrus, right hippocampus, right parahippocampal gyrus, left caudate nucleus, right inferior temporal gyrus, left olfactory cortex | 10, 32, 6, 47, 45, 44, 46, 24, 13, 11, 38, 37, 34, 28, 27, 17, 18, 20, 37 | 2320 | -15 | 18 | 18 | 5.758 |
| Cluster 3 |  |  |  |  |  |  |
| Left middle temporal gyrus, left superior temporal gyrus, left postcentral gyrus, left precentral gyrus | 21, 22, 1, 2, 3, 4 | 476 | -60 | -12 | 30 | -5.072 |
| Cluster 4 |  |  |  |  |  |  |
| Bilateral calcarine fissure and surrounding cortex, bilateral lingual gyrus, bilateral cuneus, bilateral superior occipital gyrus, right fusiform gyrus, right middle occipital gyrus, left inferior occipital gyrus | 17, 18, 37, 19, 7 | 1625 | 18 | -66 | 3 | -6.817 |
| Cluster 5 |  |  |  |  |  |  |
| Right middle temporal gyrus, right inferior temporal gyrus, right middle occipital gyrus, right inferior occipital gyrus | 21, 20, 18, 17 | 416 | 51 | -69 | -3 | -6.306 |
| Cluster 6 |  |  |  |  |  |  |
| Left middle occipital gyrus, left inferior occipital gyrus, left middle temporal gyrus, left inferior temporal gyrus | 18, 17, 21, 20 | 256 | -45 | -69 | -3 | -6.595 |
| Cluster 7 |  |  |  |  |  |  |
| Bilateral postcentral gyrus, bilateral paracentral lobule, bilateral precentral gyrus, bilateral Supplemental motor area, bilateral superior parietal gyrus, bilateral precuneus, bilateral median cingulate and paracingulate gyri, right angular gyrus | 1, 2, 3, 4, 6, 39, 40, 7, 24, 27 | 1847 | 9 | -45 | 69 | -6.795 |

*****Significant at voxel *P* value <0.01, cluster *P* value <0.05 corrected by GRF correction.

**Supplementary Table 3. ReHo differences in brain regions between vehicle rats and MAM rats in adolescence.**

| Clusters | Paxinos-Watson area number | Brain region | Cluster size | Peak MNI coordinates | | | T***** |
| --- | --- | --- | --- | --- | --- | --- | --- |
|  |  |  |  | X | Y | Z |  |
| Posterior region | 146/147 | Left auditory cortex | 3203 | -21 | -32 | -45 | 5.200 |
|  | 866 | Left temporal association cortex |  |  |  |  |  |
|  | 196/197/358/359/360/747/748 | Left hippocampus, right hippocampus |  |  |  |  |  |
|  | 456 | Left entorhinal cortex |  |  |  |  |  |
|  | 750/751/752/753/760 | Left somatosensory cortex |  |  |  |  |  |
|  | 70/73/99/126/143/163/164/167/174/203/205/206/442/447/529/537/652/658 | Left amygdala, right amygdala |  |  |  |  |  |
|  | 232/497 | Left striatum |  |  |  |  |  |
|  | 200 | Left corpus collosum |  |  |  |  |  |
|  | 104/259/341 | Left insular cortex |  |  |  |  |  |
|  | 254/895/1014/1015 | Left piriform cortex |  |  |  |  |  |
|  | 458 | Left globus pallidus |  |  |  |  |  |
|  | 366 | Left internal capsule |  |  |  |  |  |
|  | 416/418/419 | Left interstitial nucleus of the posterior limb of the anterior commissure |  |  |  |  |  |
|  | 279 | Left mesencephalic region, right mesencephalic region |  |  |  |  |  |
|  | 267 | Left periaqueductal grey, right periaqueductal grey |  |  |  |  |  |
|  | 802/806/807/808 | Left substantia nigra, right substantia nigra |  |  |  |  |  |
|  | 90/110/113/149/152/223/227/363/378/451/454/481/482/483/484/517/518/520/21580/598/627/643/648/670/672/674/698/701/702/704/743/777/797/887/898/907/920/921 | Left thalamus, right thalamus |  |  |  |  |  |
|  | 926 | Left ventral tegmental area |  |  |  |  |  |
|  | 543/546 | Right medial geniculate |  |  |  |  |  |
|  | 393/401/597/857 | Right superior colliculus |  |  |  |  |  |

*****Significant at voxel *P* value <0.05, cluster *P* value <0.05 corrected by GRF correction. Posterior region represents the cluster located in the posterior region, demonstrating significant between-group differences in adolescence.

**Supplementary Table 4. ReHo differences in brain regions between vehicle rats and MAM rats in adulthood.**

| Clusters | Paxinos-Watson area number | Brain region | Cluster size | Peak MNI coordinates | | | T***** |
| --- | --- | --- | --- | --- | --- | --- | --- |
|  |  |  |  | X | Y | Z |  |
| Frontal region | 375/691 | Left medial prefrontal cortex, right medial prefrontal cortex | 2699 | -35 | 8 | -66 | 5.071 |
|  | 266/468/560/917 | Left orbital frontal cortex, right orbital frontal cortex |  |  |  |  |  |
|  | 79 | Left accumbens nucleus core, right accumbens nucleus core |  |  |  |  |  |
|  | 441 | Left accumbens nucleus shell, right accumbens nucleus shell |  |  |  |  |  |
|  | 232/497 | Left striatum, right striatum |  |  |  |  |  |
|  | 200/330 | Left corpus collosum, right corpus collosum |  |  |  |  |  |
|  | 101/102/104/105/259/341 | Right insular cortex, left insular cortex |  |  |  |  |  |
|  | 254/895/1013/1014/1015/1016 | Right piriform cortex, left piriform cortex |  |  |  |  |  |
|  | 759/760 | Right somatosensory cortex, left somatosensory cortex |  |  |  |  |  |
|  | 458 | Right globus pallidus, left globus pallidus |  |  |  |  |  |
|  | 416 | Right interstitial nucleus of the posterior limb of the anterior commissure, left interstitial nucleus of the posterior limb of the anterior commissure |  |  |  |  |  |
|  | 116/118/119 | Right olfactory nuclei |  |  |  |  |  |
|  | 1018/1019,1020/1021 | Right olfactory tubercle |  |  |  |  |  |
|  | 493/494/498 | Septum |  |  |  |  |  |
|  | 919 | Right ventral pallidum |  |  |  |  |  |
|  |  |  |  |  |  |  |  |
| Posterior region | 882/883/884 | Left visual cortex | 3771 | -66 | -72 | -45 | 7.746 |
|  | 145/146/147 | Left auditory cortex |  |  |  |  |  |
|  | 866 | Left temporal association cortex |  |  |  |  |  |
|  | 197/359/360/673/693/746/748 | left hippocampus, right hippocampus |  |  |  |  |  |
|  | 200/331 | Left corpus collosum |  |  |  |  |  |
|  | 741 | Right retrosplenial cortex, left retrosplenial cortex |  |  |  |  |  |
|  | 458 | Left globus pallidus |  |  |  |  |  |
|  | 70/99/143/203/205/206/529/537/538/658 | Left amygdala |  |  |  |  |  |
|  | 40/41/249/459/646 | Left hypothalamus, right hypothalamus |  |  |  |  |  |
|  | 366 | Left internal capsule |  |  |  |  |  |
|  | 543/546 | Left medial geniculate |  |  |  |  |  |
|  | 279 | Right mesencephalic region, left mesencephalic region |  |  |  |  |  |
|  | 267/272/471/844 | Left periaqueductal grey, right periaqueductal grey |  |  |  |  |  |
|  | 802/806/807 | Left substantia nigra, right substantia nigra |  |  |  |  |  |
|  | 282/393/401/597/857 | Left superior colliculus, right superior colliculus |  |  |  |  |  |
|  | 110/481/483/580/598/643/648/670/672/674/716/720/743/777/847/853/856/887/898/907/920/921/924 | Left thalamus, right thalamus |  |  |  |  |  |
|  | 937/938/939 | Left zona incerta, right zona incerta |  |  |  |  |  |

*****Significant at voxel *P* value <0.05, cluster *P* value <0.05 corrected by GRF correction. Frontal region represents the cluster located in the anterior region, demonstrating significant between-group differences in adulthood; Posterior region represents the cluster located in the posterior region, demonstrating significant between-group differences in adulthood.

**Supplemental Table 5. Metabolites that enriched in central carbon metabolism in the PFC and the OC.**

| Detection method | RT  (s) | Theoretical  (m/z) | Formula | Metabolites | Class | VIP | Fold change | P value | KEGG | HMDB |
| --- | --- | --- | --- | --- | --- | --- | --- | --- | --- | --- |
| PFC |  |  |  |  |  |  |  |  |  |  |
| ESI(-) | 742.55 | 146.05 | C5H9NO4 | L-Glutamate | Amino acids | 7.78 | 0.77 | 0.003 | C00025 | HMDB0000148 |
| ESI(-) | 698.93 | 145.06 | C5H10N2O3 | L-Glutamine | Amino acids | 9.69 | 0.78 | 0.012 | C00064 | HMDB0000641 |
| ESI(-) | 969.12 | 338.99 | C6H14O12P2 | D-Fructose 1,6-bisphosphate | Carbohydrates | 4.46 | 0.37 | 0.014 | C00354 | HMDB0001058 |
| ESI(-) | 749.26 | 88.04 | C3H7NO2 | L-Alanine | Amino acids | 3.00 | 0.68 | 0.021 | C00041 | HMDB0000161 |
| ESI(-) | 777.36 | 132.03 | C4H7NO4 | L-Aspartate | Amino acids | 5.88 | 0.65 | 0.022 | C00049 | HMDB0000191 |
| ESI(-) | 755.50 | 133.01 | C4H6O5 | L-Malic acid | Beta hydroxy acids | 6.40 | 0.78 | 0.043 | C00149 | HMDB0000156 |
| ESI(-) | 799.75 | 157.12 | C3H5O6P | Phosphoenolpyruvate | Phosphate esters | 1.44 | 0.77 | 0.048 | C00074 | HMDB0000263 |
| ESI(+) | 830.16 | 147.08 | C6H9N3O2 | L-Histidine | Amino acids | 1.05 | 0.77 | 0.049 | C00135 | HMDB0000177 |
| OC |  |  |  |  |  |  |  |  |  |  |
| ESI(-) | 742.82 | 145.06 | C5H10N2O3 | L-Glutamine | Amino acids | 2.16 | 0.77 | 0.031 | C00064 | HMDB0000641 |
| ESI(-) | 596.87 | 116.07 | C5H11NO2 | L-Valine | Amino acids | 2.20 | 0.56 | 0.045 | C00183 | HMDB0000883 |
| ESI(-) | 747.82 | 132.03 | C4H7NO4 | L-Aspartate | Amino acids | 7.21 | 0.92 | 0.049 | C00049 | HMDB0000191 |
| ESI(-) | 936.47 | 191.02 | C6H8O7 | Citrate | Carbohydrates | 12.50 | 1.53 | 0.050 | C00158 | HMDB0000094 |
| ESI(+) | 762.50 | 156.08 | C6H9N3O2 | L-Histidine | Amino acids | 8.06 | 0.70 | 0.048 | C00135 | HMDB0000177 |

RT, retention time; VIP, variable importance in projection; KEGG, Kyoto Encyclopedia of Genes and Genomes; HMDB, Human Metabolome Database; ESI, Electrospray ionization.

**Reference**

1. Nozari, M., Mansouri, F.A., Shabani, M., Nozari, H. & Atapour, N. Postnatal MK-801 treatment of female rats impairs acquisition of working memory, but not reference memory in an eight-arm radial maze; no beneficial effects of enriched environment. *Psychopharmacology* **232**, 2541-2550 (2015).

2. Nozari, M., Shabani, M., Hadadi, M. & Atapour, N. Enriched environment prevents cognitive and motor deficits associated with postnatal MK-801 treatment. *Psychopharmacology* **231**, 4361-4370 (2014).

3. Swanson, L.W. Brain maps 4.0-Structure of the rat brain: An open access atlas with global nervous system nomenclature ontology and flatmaps. *The Journal of comparative neurology* **526**, 935-943 (2018).
